# Editorial: Enzyme targeting for the development of novel antimicrobials

**DOI:** 10.3389/fmolb.2024.1472318

**Published:** 2024-08-19

**Authors:** Elena G. Salina, Chunhua Qiao, Laurent R. Chiarelli

**Affiliations:** ^1^ Bach Institute of Biochemistry, Research Centre of Biotechnology RAS, Moscow, Russia; ^2^ College of Pharmaceutical Sciences, Soochow University, Suzhou, China; ^3^ Department of Biology and Biotechnology, University of Pavia, Pavia, Italy

**Keywords:** antimicrobials, antimicrobial resistance, enzymes, enzymatic targets, antimicrobial targets

The spread of antimicrobial resistance (AMR) has become a major threat to global health and healthcare systems, being the cause of at least 700,000 deaths per year worldwide, a figure that is estimated to reach 10 million by 2050 if effective action is not taken. In all likelihood, the misuse and overuse of antimicrobial agents in healthcare, agriculture, and livestock are the main causes of the spread of AMR; therefore, combating AMR requires not only different approaches, such as improved antibiotic stewardship, but also the development of new antimicrobials and vaccines, along with the use of alternative strategies. The urgent need for new and improved antimicrobial drugs implies the need to identify new targets and new molecular structures to overcome cross-resistance. The objective of this Research Topic “Enzyme targeting for the development of novel antimicrobials” is to gather recent advances in the investigation of potential enzyme targets, which are involved not only in essential microbial metabolic pathways but also in pathways crucial for the establishment and maintenance of infection, as well as host enzymes in the context of host-directed therapy.

This latter approach is the topic of the paper by Konaklieva and Plotkin. This review article presents an overview of host lipid metabolism and its connections with the development of host-directed therapies for the treatment of viral and bacterial infections. Indeed, several pathogens can utilize host lipid enzymes or produce their own analogs, thus interfering with human host lipid metabolism.

The second paper by Roszczenko-Jasińska et al. presents a more classical structure-assisted drug design approach to develop peptide-based inhibitors against *H. pylori* DsbK, a periplasmic oxidoreductase involved in disulfide bond formation. Interestingly, Dsb proteins are involved in virulence pathways such as adhesion and motility, which are essential for establishing infections but not essential for bacterial growth. In this work, three synthetic peptides were designed and proved to effectively inhibit DsbK oxidase activity, providing the basis for a new targeted strategy against *Helicobacter pylori*, which deprives the bacterium of its pathogenic properties without compromising viability, and does not influence the natural human microbiome. This anti-virulence approach does not exert selective pressure, thus conceivably reducing the risk of resistance phenomena.

Computational tools are becoming increasingly important in the drug discovery process, as is the case in the work of Nazim et al. In this work, a computational modeling approach was used to perform a virtual high-throughput screening of a library of Food and Drug Administration-approved drugs against the *M. tuberculosis* RNA methyltransferase Rv3366 structure. This approach of repurposing existing drugs is a strategy that seems promising for addressing the problem of antimicrobial resistance as the use of already approved drugs can accelerate transposition into the clinical setting. This multi-step structure-based drug repurposing workflow allowed the identification of levodopa and droxidopa as promising Rv3366 inhibitors, confirming the importance of targeting RNA methyltransferases to combat drug-resistant *Mycobacterium tuberculosis* and proposing levodopa and droxidopa as new leads for future preclinical investigation.

In some cases, it may not be easy to identify the target of antimicrobial molecules, especially if they actually have a primary mechanism of action different from classical enzyme inhibition. This is the case of the work of Fontes et al., which investigated the mechanism of action of pyrazinoic acid. Pyrazinoic acid is the active form of pyrazinamide, a first-line antimycobacterial drug, whose mechanism of action remains a subject of debate. In this work, the authors demonstrated that pyrazinoic acid exhibits a pH-dependent inhibition of mycobacterial growth, similar to other known protonophores and unlike other antitubercular drugs. Furthermore, the quantitative structure–activity relationship model used indicated that this inhibition depends exclusively on the concentration of the protonated form of this weak acid, rather than the deprotonated form. These results, therefore, led to the conclusion that pyrazinoic acid acts as an uncoupler of oxidative phosphorylation through disruption of the proton motive force, contributing to a better understanding of the mechanism of action of this important first-line drug.

As already mentioned, antimicrobials are not only small molecules but can also be peptides or even proteins. In this context, Fernández-Millán et al. Present a new protein-based strategy for alternative therapies. The authors focused on the RNase A superfamily, in particular on an RNase chimera that combines the high catalytic activity of RNase 1 with specific antimicrobial regions of RNase 3. Through a crystallographic study of three versions of this RNase 3/1 chimera, the authors identified key determinants explaining the specific properties of each variant, obtaining useful information to guide the design of the best RNase pharmacophore for antimicrobial therapies.

Overall, this Research Topic provides an overview of the most recently applied approaches in the antimicrobial drug discovery process. These include classical target-to-drug strategies, implemented by computational tools to accelerate the development of antibacterial or antivirulence compounds, whether small molecules or peptides or even proteins, as well as to identify new uses of already approved drugs in the context of the repurposing strategy.

